# Long-term continuous infusion of anti-GD2 antibody CH14.18/CHO in relapsed/refractory neuroblastoma patients

**DOI:** 10.1186/2051-1426-1-S1-P244

**Published:** 2013-11-07

**Authors:** Holger Lode, Nikolai Siebert, Silke Kietz, Ina Müller, Manfred Schuster, Evelyne Janzek, Susanne Wiederkum, Oliver Mutschlechner, Hans Loibner

**Affiliations:** 1 Pediatric Hematology and Oncology, University Medicine, Greifswald, Germany; 2 Apeiron Biologics, Vienna, Austria

## Purpose

GD2 is highly expressed in neuroblastoma (NB) and specifically recognized by antibodies ch14.18. Immunotherapy with bolus infusion of ch14.18 in combination with cytokines prolonged survival in patients at risk for relapse but was associated with substantial toxicity, in particular severe pain, requiring i.v. morphine [EJM 2010]. In an attempt to reduce the pain side effect associated with anti-GD2 therapy and to optimize antibody exposure, we piloted a treatment using continuous infusion of ch14.18 produced in CHO cells (APN311) and report first results.

## Materials and methods

Relapsed or refractory NB patients received 5 cycles of 6x10^6^ IU/m^2^ s.c. IL-2 (d1-5; 8-12), 10 day continuous infusion of 100 mg/m^2^ APN311 (d8-17) and 160 mg/m^2^ oral 13-cis-RA (d19-32). The cytolytic potential of antibody treatment was assessed using a whole blood test (WBT) and by CDC. For WBT, fresh heparinized whole blood of patients was used as effector for lysis of 51Cr-labeled human GD2+ LAN1 NB cells. Antibody specificity of lysis was determined by adding excess of an anti-idiotypic antibody to APN311 (ganglidiomab). APN311 levels in sera were determined by ELISA using ganglidiomab. Pain assessment during APN311 infusions was done by standardized evaluation methods and by reduction / complete avoidance of i.v. morphine. Clinical responses were assessed in particular by MIBG scintigraphy.

## Results

All analyzed patients demonstrated GD2 specific lysis in their blood and serum against LAN1 NB cells. This lytic activity was consistently high during all long term antibody infusions. In > 70% of patients the lytic activity of whole blood in later cycles remained significantly above base line until start of the next antibody infusion, thereby creating a continuous lytic potential for several months. Pain assessment showed that 80% of patients after 5 days antibody infusion already in cycle 1 did not require i.v. morphine anymore, and in later cycles there was almost no need for i.v. morphine. Clinical response rates > 30% based on MIBG evaluation were seen.

## Conclusion

5 cycles of a 10 day continuous infusion of APN311 (in conjunction with s.c. IL2 and oral 13-cis-RA) in relapsed/refractory NB patients revealed a high and sustained APN311-specific lytic potential against GD2+ NB cells in whole blood. The pain side effect was substantially reduced, thereby almost completely avoiding i.v. morphine co-medication. Response rates of > 30% were observed suggesting clinical activity of this novel treatment modality. Confirmatory clinical trials are warranted to substantiate these first results.

